# Molecular Mechanisms of Resistance against PSII-Inhibiting Herbicides in *Amaranthus retroflexus* from the Czech Republic

**DOI:** 10.3390/genes15070904

**Published:** 2024-07-11

**Authors:** Jakub Mikulka, Madhab Kumar Sen, Pavlína Košnarová, Pavel Hamouz, Kateřina Hamouzová, Vishma Pratap Sur, Jaromír Šuk, Soham Bhattacharya, Josef Soukup

**Affiliations:** 1 Department of Agroecology and Crop Production, Faculty of Agrobiology, Food and Natural Resources, Czech University of Life Sciences Prague, Kamýcká 1176, 165 00 Prague, Czech Republic; mikulkajakub@af.czu.cz (J.M.); senm@af.czu.cz (M.K.S.); kosnarova@af.czu.cz (P.K.); hamouzp@af.czu.cz (P.H.); hamouzova@af.czu.cz (K.H.); sukjaromir@af.czu.cz (J.Š.); bhattacharya@af.czu.cz (S.B.); 2 Institute of Microbiology, The Czech Academy of Sciences, Centre Algatech, Novohradská 237-Opatovický Mlýn, 379 01 Třebon, Czech Republic; sur@alga.cz

**Keywords:** D1 protein mutations, molecular docking, chlorophyll fluorescence

## Abstract

*Amaranthus retroflexus* L. (redroot pigweed) is one of the most problematic weeds in maize, sugar beet, vegetables, and soybean crop fields in Europe. Two pigweed amaranth biotypes (R1 and R2) from the Czech Republic resistant to photosystem II (PSII)-inhibiting herbicides were analyzed in this study. This study aimed to identify the genetic mechanisms that underlie the resistance observed in the biotypes. Additionally, we also intended to establish the use of chlorophyll fluorescence measurement as a rapid and reliable method for confirming herbicide resistance in this weed species. Both biotypes analyzed showed high resistance factors in a dose–response study and were thus confirmed to be resistant to PSII-inhibiting herbicides. A sequence analysis of the D1 protein revealed a well-known Ser-Gly substitution at amino acid position 264 in both biotypes. Molecular docking studies, along with the wild-type and mutant D1 protein’s secondary structure analyses, revealed that the S264G mutation did not reduce herbicide affinity but instead indirectly affected the interaction between the target protein and the herbicides. The current study identified the S264G mutation as being responsible for conferring herbicide resistance in the pigweed amaranth biotypes. These findings can provide a strong basis for future studies that might use protein structure and mutation-based approaches to gain further insights into the detailed mechanisms of resistance in this weed species. In many individuals from both biotypes, resistance at a very early stage (BBCH10) of plants was demonstrated several hours after the application of the active ingredients by the chlorophyll fluorescence method. The effective PS II quantum yield parameter can be used as a rapid diagnostic tool for distinguishing between sensitive and resistant plants on an individual level. This method can be useful for identifying herbicide-resistant weed biotypes in the field, which can help farmers and weed management practitioners develop more effective weed control tactics.

## 1. Introduction

For more than two decades, weeds have not been exposed to herbicides with new modes of action. This had led to a serious scenario, where reports suggest that weeds have gradually developed resistance to almost 65% of the known herbicide sites of action (https://www.weedscience.org/Home.aspx, accessed on 10 April 2024). *Amaranthus retroflexus* L. (redroot pigweed) is one of the most problematic C4 species weeds in crops worldwide, becoming also more and more important in Europe. This species is difficult to manage mainly because of abundant seed production, extended germination periods, long seed viability, and annual dormancy variability [1,2]. Herbicides are widely and extensively used against *Amaranthus* species. To date, ~11 *Amaranthus* species including *A. retroflexus* and other closely related species like *A. hybridus* and *A. tuberculatus* have been reported to be involved in resistance worldwide [3,4,5]. Currently, in Europe, pigweed amaranth and its related species are commonly controlled using photosystem II (PSII)-inhibiting, acetolactate synthase (ALS)-inhibiting, and protoporphyrinogen oxidase (PPO)-inhibiting herbicides. However, ever since the discovery of the first herbicide-resistant *A. retroflexus* biotype in Austria (1973), resistance against several other PSII-inhibiting, ALS-inhibiting and PPO-inhibiting herbicides have also been reported across the globe [6,7,8,9]. Recently, the widespread use of herbicides has led to the evolution of herbicide resistance in pigweed amaranth species in central Europe, where PSII inhibitors are mainly used, to control this species. Although herbicide resistance can be either target-site-based (TSR) or non-target-site-based (NTSR), the evolution of herbicide resistance against PSII-inhibiting herbicides in weeds is typically TSR-based. PSII-inhibiting herbicides such as atrazine, terbuthylazine, and metamitron are known to compete with QB at the QB-binding site in the D1 protein, which finally leads to the inhibition of NADPH and ATP production. Finally, the plants die due to carbohydrate starvation and oxidative stress [9]. Even though over 30 single amino acid substitutions within the D1 protein have been reported in cyanobacteria, algae, and higher plants, only eight of these mutations are identified to confer resistance in arable weedy species. These eight mutated positions include Leu218, Val219, Ala251, Phe255, Ser264, Asn266, Phe274, and Leu275 (numbering based on the GenBank accession number ABI54129.1) [10,11,12,13,14,15,16,17]. Among these eight, Ser264Gly is the most common PSII-inhibiting herbicide resistance-endowing mutation. Additionally, NTSR mechanisms such as enhanced metabolism can also bestow resistance to these groups of herbicides in weeds [18,19]. Since their development in the early 1980s, molecular docking studies have become a compelling part of many scientific discoveries [20]. The increasing availability of software which can predict protein structures efficiently has entrenched molecular docking studies as an indispensable part of compound discovery with novel properties. An in-depth understanding of the binding patterns of a ligand, along with the identification of interactive sites between ligand molecules and proteins of interest, is crucial for the rational design of effective herbicidal compounds. Although some molecular modeling and docking-based studies with herbicide-targeting proteins and herbicidal compounds at the molecular level have been conducted to date [21,22,23], many more such studies are needed to understand the more detailed effects of the mutations endowing herbicide resistance. In recent years, terbuthylazine and metamitron have failed to control *A. retroflexus* in the fields of the Czech Republic. Hence, the main aim of this study is to characterize the level of PSII-inhibiting herbicide resistance in two resistant redroot pigweed populations and to identify the molecular basis of resistance. Additionally, an integrated theoretical study using homology modeling and molecular docking was performed to determine if the associated *psbA* mutation can alter the conformation of the D1 enzyme and affect its binding with herbicides. Finally, chlorophyll fluorescence measurements are proven to be useful for the identification of resistant individuals to PSII inhibitors in populations of this species at early growth stages. The main goal of the chlorophyll fluorescence measurement experiment is to utilize chlorophyll fluorescence measurement as a fast and effective assay to detect herbicide resistance in individuals before visible symptoms appear. This method also aims to determine if fluorometry can more quickly and accurately identify the sub-lethal effects of herbicides on photosynthesis than traditional visual assessments. Successfully implementing this approach could improve early resistance management and lead to more efficient resistance management.

## 2. Materials and Methods

### 2.1. Plant Materials and Dose–Response Experiment

The seeds of the susceptible reference (S) biotype were collected from the campus of the Czech University of Life Sciences, Prague (50.13179 N, 14.36928 E), from a locality without previous herbicide application. The seeds of resistant biotypes R1 and R2 were collected in the Hradec Kralove region (50.30993 N, 16.10351 E) and South Moravian region (48.82358 N, 16.18497 E) from fields of sugar beet and maize where PSII inhibitors metamitron and terbuthylazine were applied. Thereafter, the seeds were sown in pots (250 mL, filled with chernozem soil containing high fertility properties and moisture storage capacity). Emerged seedlings were tinned to ten plants/pots. A dose–response test was conducted under unheated cover-top greenhouse conditions from April to June in a completely randomized design with four replicates. The seedlings were regularly watered, and fertilizers were applied as required. At the cotyledon stage (BBCH 10), the plants were treated in eight increasing doses listed in Table 1. Plants treated with water served as an untreated control. A laboratory spray chamber, equipped with a Lurmark 01F80 nozzle, was calibrated to the spray volume of 250 L ha^−1^ at a spraying pressure of 250 kPa. The above ground biomass of all treatments was harvested to determine the production of shoot dry weight. Dry weight was recorded after drying the plants at 65 °C in an oven for 48 h when the plants were completely dried and free of moisture.

### 2.2. Statistical Analysis

Dose–response experiments were conducted to estimate an effective dose reducing 50% of growth (ED50) compared to the untreated control of a respective biotype and resistance factors (RFs). RF was calculated as the ratio of the ED50 values of resistant and susceptible standard populations. For fitting the log-logistic curves, four parameters models were used. The dose–response curves were plotted by a non-linear regression model using the drc package R-Studio program (https://www.r-project.org/ accessed on 10 April 2024). The heterogeneity adjustment was performed through a Box–Cox transformation. The quality of each set of dose–response models was compared with an analysis of variance by a lack-of-fit F-test (ANOVA—built-in function in the drc package).

### 2.3. psbA Gene Partial Sequencing

A total of 30 leaves from each biotype were collected from surviving plants after metamitron and terbuthylazine application and sent to Jealott’s Hill, Syngenta, the UK, for sequencing. Shock-frozen leaf tissues (±80 mg per sample) from R and S biotypes were used for genomic DNA (gDNA) extraction using the DNeasy Plant Mini Kit (QIAGEN, Hilden, Germany) following the manufacturer’s instructions. Sanger sequencing was performed on these leaf samples around the psbA gene using gene-specific forward 5′ GCTATTATTCCTACTTCTGC 3′ and reverse 5′ CCATTTGTAGATGGAGC 3′ primers. The amplicon contained the following mutation points: 211, 219, 250, 251, 255, 256, 257, 263, 264, 266, 268, 269, 275, and 282. The sequencing results were aligned and analyzed in BioEdit (version 7.2).

### 2.4. 3D Modeling and Molecular Docking Studies

SWISS-MODEL (https://swissmodel.expasy.org/ accessed on 10 April 2024) was used to predict the 3D structures of the wild-type and mutant-type D1 protein (Ser264Gly) from redroot pigweed. The most appropriate homology-modeled structure was chosen based on the sequence identity with the template, the Global Model Quality Estimate (GMQE), the QMEANDisCo global score, and the percentage of Ramachandran-favored scoring functions. All the predicted 3D structures were visualized by UCSF Chimera 1.15rc software. To begin the molecular docking studies, the chemical structures of the herbicides were chosen from PubChem, a database of chemical molecules, maintained by the National Center for Biotechnology Information in the United States, freely available at https://pubchem.ncbi.nlm.nih.gov/ accessed on 10 April 2024. The 3D structures of the herbicides were retrieved from PubChem in an SDF file format, which were further converted into a PDB file format. These PDB files were used in the PyRx 0.8 Autodock Vina module, a molecular docking and virtual screening program. Then, mgltools was used for the molecular interaction studies. Finally, AutoDock Vina 1.1.2 in PyRx 0.8 software (ver.0.8, Scripps Research, La Jolla, CA, USA) was used for molecular docking [24].

### 2.5. Chlorophyll Fluorescence Measurement

Seeds of R1 and R2 biotypes were sown into in pots (8 × 8 × 8 cm^3^). After germination, seedlings of uniform size were spread across five plants per pot. Terbuthylazine and metamitron were applied at recommended field doses at the cotyledon stage (BBCH 10) of redroot pigweed. The experiment was designed with four replicates. A total of 20 randomly selected plants were measured for each biotype. The fluorescence measurements were performed using the pulse amplitude modulation fluorometer Imaging-PAM Maxi (Walz, Germany) and Imaging Win software version V2.41. The cotyledon leaves of each plant were used for chlorophyll fluorescence measurements after 10 min of dark adaptation by covering the plants with a non-translucent box at room temperature. The fluorescence slow induction curve of the intact leaves was measured immediately before herbicide treatment. This measurement represents time zero in the charts. After herbicide application, the measurements were taken at the following times: 1 h, 3 h, 6 h, 12 h, 24 h, 2 d, 3 d, 4 d, 6 d, 8 d, and 10 days after treatment (DAT) of the single plants on an individual basis. At the beginning of each measurement, a saturation pulse was provided to measure the minimal (F0) and maximal (Fm) chlorophyll fluorescence. Maximal PS II quantum yield Fv/Fm was calculated according to the following equation:

Fv/Fm = (Fm − F0)/Fm (1)

Slow induction kinetics curves were determined by using automated programs provided by the Imaging Win software version 2.47. Forty seconds after the saturation pulse, the actinic light was turned on and 10 saturation pulses were given in 20 s intervals. The saturation pulse had an intensity of 6000 µE m^−2^ s^−1^ at an emission peak of 450 nm. Changes in fluorescence parameters were recorded automatically involving a total of 10 measurements over each treatment. The value of effective PSII quantum yield (QY PSII) after 10 saturation pulses was used for a comparison of the photosynthetic activity of individual plants. The effective PSII quantum yield was calculated according to Genty et al. (1989) by the following formula [25]:

Y(II) = (Fm′ − F)/Fm′ (2)

Explorative data analysis followed by ANOVA at the α = 0.05 probability level was performed in software Statistica 12 [26].

## 3. Results

### 3.1. Dose–Response Test

The results of the dose–response experiment proved that both *A. retroflexus* biotypes evolved a high-level of resistance to PSII-inhibiting herbicides. The GR50 value reached 5512.5 g a.i. ha^−1^ of terbuthylazine for R1, while a dose of 45.75 g a.i. ha^−1^ was needed to achieve a 50% growth reduction in the susceptible reference biotype. This resulted in an RF value of 120.5, while the active ingredient metamitron resulted in an RF value of 3.3.

**Figure 1:**
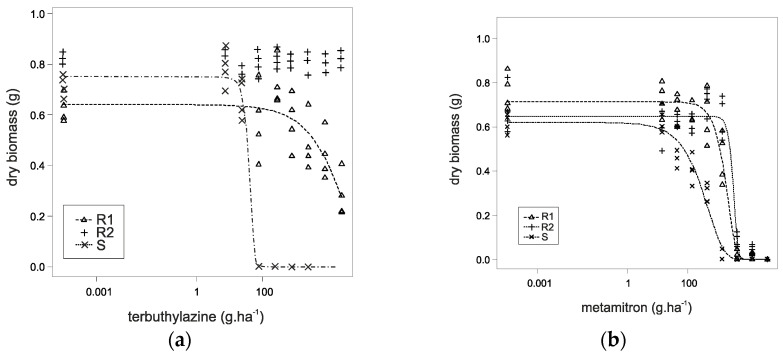
Fitted logarithmic dose–response curves for *A. retroflexus* (**a**) with terbuthylazine and (**b**) with metamitron. S: susceptible biotype; R1 and R2 are the resistant biotypes. In the case of R2, it was not possible to draw the dose–response curve for terbuthylazine, due to high values (for the detailed data, please refer to Table 2).

The biotype R2 survived nearly unaffected by the application of terbuthylazine at all doses. A dose higher than 23,700 g a.i. ha^−1^ was needed to achieve a 50% growth reduction and RF values were higher than 518. For metamitron, the RF value was 4.4 for R2 (Table 2). The dose–response curves are shown in Figure 1.

### 3.2. Sequencing of psbA Gene from A. retroflexus

**Figure 2:**
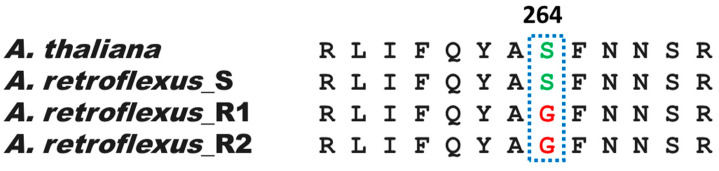
Alignment of the partial D1 protein sequences for mutation analysis.

From the S and R biotypes, 818 bp of the amaranth chloroplast psbA gene was amplified and sequenced. The obtained gene sequence was then translated and aligned with the Arabidopsis thaliana orthologue. This included the amino acid region of the D1 protein which contained the eight most significant mutated positions (Leu218, Val219, Ala251, Phe255, Ser264, Asn266, Phe274, and Leu275). A comparison between susceptible (S) and resistant (R) biotypes showed that the only mutation detected was the well-known Ser264Gly (S264G), as seen in Figure 2. No mutations were detected for the S biotype. Among the tested plants, 90% in biotype R1 and 100% in biotype R2 exhibited this mutation, with no other mutations observed.

### 3.3. Three-Dimensional Structural Modeling of the Wild-Type and the Mutant-Type D1 Protein variants and Molecular Docking Experiments

In most species, the resistance to PSII-inhibiting herbicides, such as triazines and triazinones, is mainly due to the substitution of glycine for serine at the 264th position of the D1 protein. The effects and impacts of mutations might vary from being beneficial to having lethal or no consequences. Hence, to investigate the actual mechanisms of PSII-inhibiting herbicides, we performed molecular docking analysis. Prior to this, we had predicted the best three-dimensional structures of the wild-type and the mutant D1 protein based on the parameters shown in Table 3.

**Figure 3:**
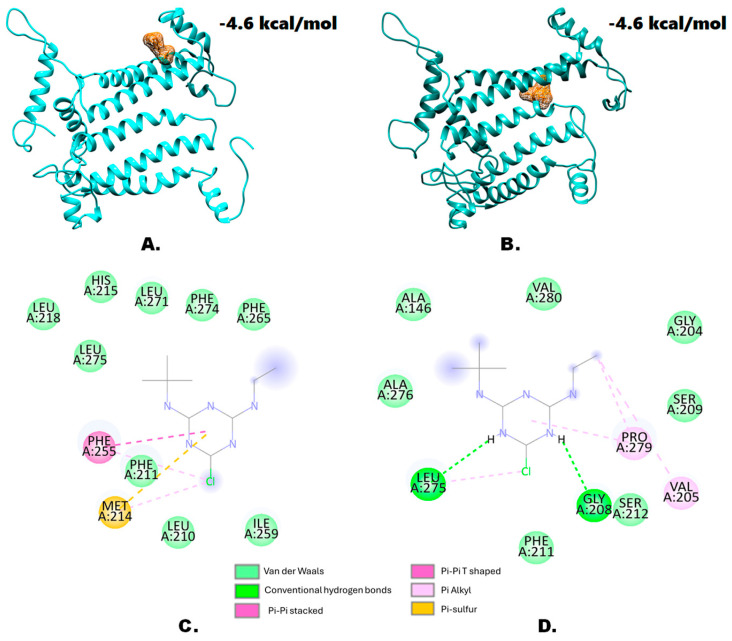
Molecular docking analysis results with terbuthylazine. Predicted structure of the (**A**) wild-type D1 protein and (**B**) mutant-type D1 protein (Ser264Gly) modeled through homology modeling using SWISS-MODEL and visualized through the UCSF Chimera 1.15rc visualization tool. (**C**,**D**) provide a general overview of the protein–ligand interactions, depicting the amino acid residues involved from the wild-type D1 protein and the mutant D1 protein (Ser264Gly), respectively.

**Figure 4:**
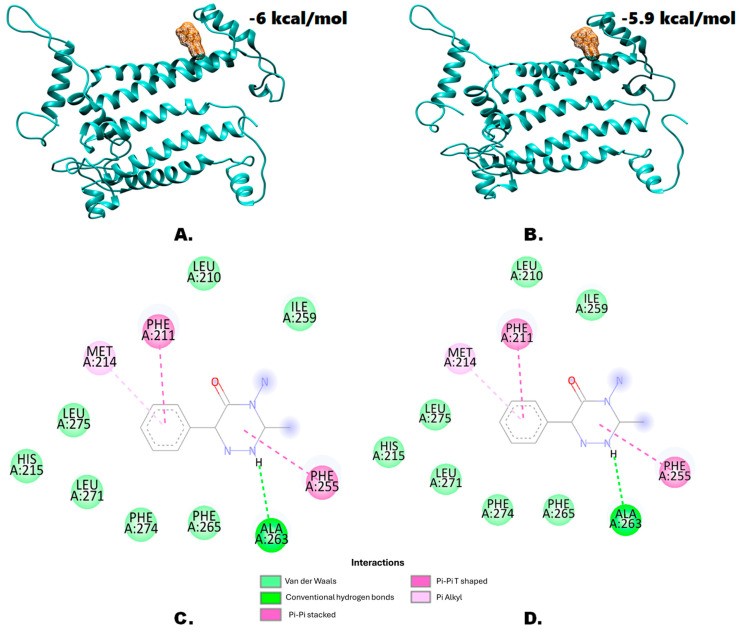
Molecular docking analysis results with METAMITRON. Predicted structure of the (**A**) wild-type D1 protein and (**B**) mutant-type D1 protein (Ser264Gly) modeled through homology modeling using SWISS-MODEL and visualized through the UCSF Chimera 1.15rc visualization tool. (**C**,**D**) provide a general overview of the protein–ligand interactions, depicting the amino acid residues involved from the wild-type D1 protein and the mutant D1 protein (Ser264Gly), respectively.

Molecular docking analysis revealed the binding affinity values. No significant to a very slight change in the binding affinity values were found in the case of terbuthylazine (Figure 3A,B) and metamitron (Figure 4A,B), respectively. This indicates that despite the mutation within the target protein, reduced herbicide affinity is not the main mechanism. However, some other factors such as amino acid residues, secondary structural features, interacting bonds, etc., might be involved in imparting resistance. From the molecular docking experiments, the major amino acids and their respective bonds have been predicted for terbuthylazine (Figure 3C,D) and metamitron (Figure 4C,D). In the case of metamitron, no differences in amino acid residues and the involved bonds were detected for the wild and mutant types. Both the wild type and the mutant type bind to metamitron by one hydrogen bond, seven van der Waals bonds, and one Pi-alkyl, Pi-Pi stacked, and Pi-Pi T-shaped bond each. Hence, in this case, the elucidation of the exact mechanism might need further analysis with the protein secondary structures. Additionally, the interacting amino acids were also found to be different from those involved in the wild type. In the case of the mutant type, the involved amino acids were mostly hydrophobic but also included a hydrophilic Ser residue. However, in the case of the wild type, even though most of the amino acid residues were hydrophobic, no hydrophilic amino acids were detected. Instead, the basic amino acid His was detected. Hence, in addition to the hydrogen bonds, the hydrophilic serine at the 212th position might also play an important role in conferring resistance in *A. retroflexus*.

### 3.4. Effects of the S264G Mutation on Protein Structural Features

**Figure 5:**
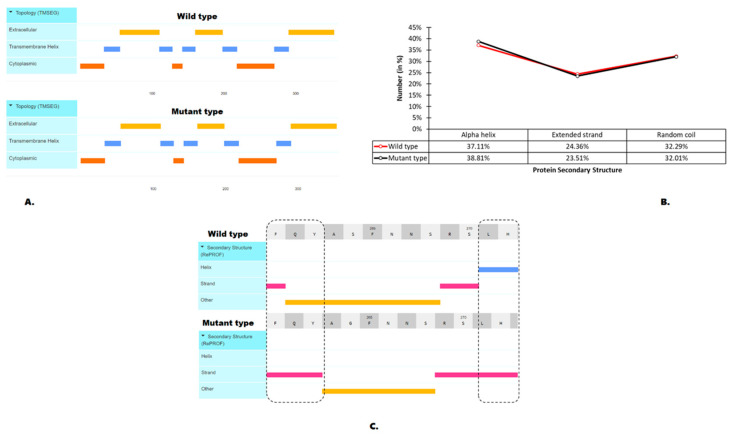
Effect of S264G mutation on the protein features. (**A**) the effect of mutation on transmembrane helix prediction, (**B**) secondary structure analysis of the wild-type and mutant-type D1 protein, (**C**) the effect of mutation on the prediction of secondary structure and solvent accessibility and secondary structure analysis of the wild-type and mutant-type D1 protein.

No significant differences in the D1 protein topology were detected between the wild type and the mutant type (Figure 5A). However, from the overall secondary structure analysis, slight differences were identified (Figure 5B). Furthermore, a more detailed secondary structure analysis around the mutation points of interest (from the 260th to 272nd amino acid residues) discovered changes in the protein secondary structure around that region (Figure 5C). Hence, we can conclude that there are no direct effects of the S264G mutation on the binding of D1 protein with metamitron.

### 3.5. Chlorophyll Fluorescence Measurement

**Figure 6:**
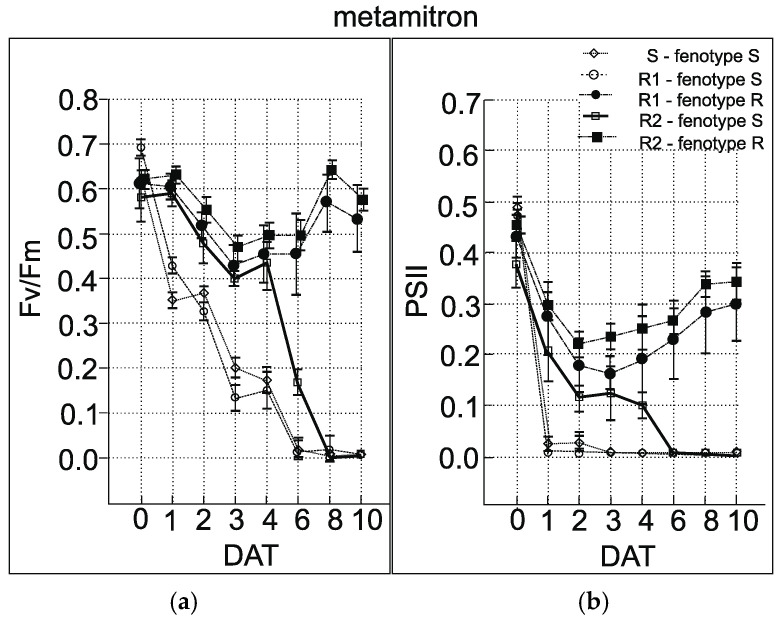
Chlorophyll fluorescence parameters Fv/Fm (**a**) and QY PS II (**b**). Points represent mean values and vertical bars are confidence intervals (95%) measured for 20 individuals of *A. retroflexus* treated with metamitron. The x axis represents days after the treatment (DAT).

**Figure 7:**
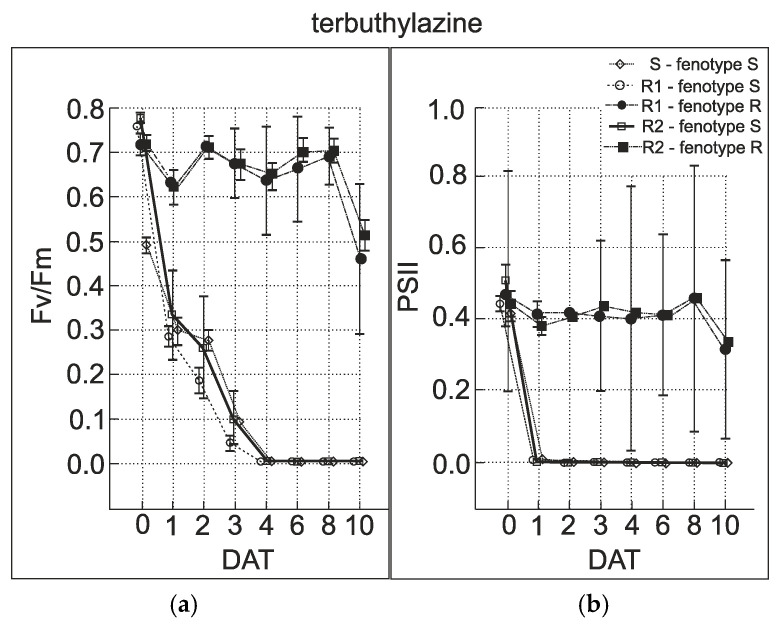
Chlorophyll fluorescence parameters Fv/Fm (**a**) and QY PS II (**b**). Points represent mean values and vertical bars are confidence intervals (95%) measured for 20 individuals of *A. retroflexus* treated with metamitron. The x axis represents days after the treatment (DAT).

The fluorescence slow induction measurements showed significant differences in fluorescence parameters between the sensitive and resistant plants of *A. retroflexus*. In the case of biotype R1, 2 of the 20 plants showed a moderate temporal decrease in QY PSII after terbuthylazine application, which was followed by an increase to nearly original levels (0.4) after 12 h. These plants were phenotypically categorized as “resistant”. The remaining plants showed rapid decrease in QY PSII similar to the sensitive populations. After 6 h, the values of QY PSII dropped to 0.1–0.02 (Figure 6). In biotype R2, the majority of plants (17 of 20) showed a moderate temporal decrease in QY PSII, whereas the other plants exhibited a decrease similar to the sensitive populations after 24 h. In the case of metamitron stress, 6 of 20 plants from R1 showed a moderate temporal decrease in QY PSII 3–6 days after the metamitron application, which was followed by gradual increase to nearly original levels after 10 days. All other plants showed a more rapid decrease in QY PSII to zero, which was similar to the sensitive population. In biotype R2, the majority of the plants (19 of 20) showed a moderate temporal decrease in QY PSII 2–3 days after metamitron application (Figures S1 and S2). This was then followed by a gradual increase to near-original levels after 10 days. One plant exhibited a gradual decrease to zero and no recovery was observed. In both the R1 and R2 population, several plants were relatively unaffected by the terbuthylazine and metamitron treatment. This supports the finding that target-site-resistant plants are present in these populations. A decrease in the parameter Fv/Fm was observed after the herbicide treatment later than the QY PSII parameter (*p* = 0.001). Especially in the case of metamitron, differences between phenotypically sensitive and resistant individuals in the first days after the treatment were insignificant (*p* < 0.05). Clear differences were first noted 5 days after application in the case of metamitron and 3 days in terbuthylazine, respectively (Figure 7). A decrease in QY PSII was observed within 60 min after herbicide application in sensitive individuals. In this regard, when using the prompt chlorophyll fluorescence technique to reveal resistance to PSII-inhibiting herbicides, we recommend the QY PSII parameter rather than Fv/Fm.

## 4. Discussion

Substantial resistance was developed by both *A. retroflexus* biotypes to terbuthylazine and metamitron, with R2 showing particularly high resistance levels. Similar results from a dose–response test studying resistance to metamitron are reported by Adamczewski et al., 2019 [27]. In three biotypes of *A. retroflexus,* the following RF values were found: 4.4, 2.9, and 2.4. Another study, however, reports high efficacy and no resistance of *A. retroflexus* after treatment with terbuthylazine, with a resistance factor (RF) close to 1 [28]. High levels of resistance to other active substances from the group of PSII inhibitors, such as bentazon, were found in *A. retroflexus*, with resistance indices of 9.01 and 6.85 for the R1 and R2 biotypes, respectively [29]. Very high GR50 and RF values can indicate the presence of target-site resistance to PS II herbicides usually caused by a TSR mutation of the psbA gene (Ser-264-Gly) [30]. Chlorophyll fluorescence measurement experiments have been carried out in many weedy species such as *Apera spica-venti* [31], *Xanthium strumarium* [32], *Solanum nigrum* [33], *Echinochloa crus-galli* [34], *Stellaria media* [35], *Papaver rhoeas* [35], etc. In these species, the Fv/Fm parameter has detected the effects of PSII-inhibiting herbicides successfully. The effect of herbicides with the active ingredients bentazone, mesotrione, and terbuthylazine on *A. retroflexus* was detected quicker by QY PSII than by any other parameters [36]. On the other hand, Wang et al. 2018 showed significant differences in the Fv/Fm parameter 1 DAT of *A. myosuroides* with PSII-inhibiting herbicides [36]. Atrazine (a PSII inhibitor) was verified to produce a significant inhibitory effect on photosynthesis, markedly depress the quantum yield, and block electron transport, thereby increasing chlorophyll a fluorescence yield and reducing O₂ evolution in the susceptible biotype (S). The resistant biotype *A. retroflexus* behaved like untreated plants in their study. Similar results were also found from our study. Since its discovery, S264G has emerged as one of the prevalent mutations for resistance to PSII-inhibiting herbicides across the world. This amino acid exchange has been found to influence the effectiveness of PSII-inhibiting herbicides in many species including *Brassica napus*, *A. retroflexus*, *A. cruetus,* and *A. hybridus* [37]. Additionally, previous studies also suggest that this mutation is known to confer high-level resistance to the triazine and triazinones herbicides but only moderate to no resistance to other chemical families, such as the ureas [16,38]. This might be the reason for the levels of resistance in the case of terbuthylazine (triazine) but not for metamitron (triazinones). The varying frequency of the TSR mutation could explain the differences in herbicide responses observed in the pots. However, it is also important to consider that NTSR mechanisms might also play a role. NTSR might involve changes in the plant’s metabolism that increase the detoxification of the herbicide, alterations in herbicide sequestration, or changes in the uptake and translocation of the herbicide within the plant. These mechanisms could contribute independently or alongside TSR to the overall resistance the phenotype showed.

Although a few molecular docking studies with wild-type and mutated D1 proteins and PSII-inhibiting herbicides have been conducted [39,40], many more such studies with target-site proteins from different organisms are needed in the future to better understand the detailed effects. Understanding the impacts of the target-site mutations will help design more effective herbicide management strategies. Molecular docking analysis revealed only negligible to slight changes, suggesting that mutations within the protein did not significantly affect herbicide affinity. This indicates that reduced herbicide affinity might not be the primary mechanism of resistance. For both herbicides, detailed analysis also showed no differences in the binding interactions (especially the hydrogen bonds) between the wild-type and mutant proteins. Hydrogen bonds are known to be responsible for most of the directional interactions involved in protein folding, protein structure, and molecular recognition [41,42]. However, while the overall protein topology did not change, slight variations in secondary structures around the mutation sites were detected. This suggests that secondary structural features, rather than direct binding affinities, might influence herbicide resistance. Differences in amino acid types and their properties, such as the presence of hydrophilic serine in the mutant versus a basic histidine in the wild type, could impact the interaction dynamics. Nevertheless, further mutational studies and plant genetic transformation studies will be required to confirm such findings. Although docking-based studies are an ineludible part of pharmaceutical research, these studies in the field of weed science are yet to gain their momentum. Molecular docking studies with the homology-modeled proteins of interest from the non-model species and the existing herbicides can serve as an important prototype study. Combined with these computational approaches, homologous protein expression studies (with optimal codons) can produce interesting results that might lead to the discovery of new compounds with herbicidal properties.

## 5. Conclusions

Herbicide resistance in two biotypes of *A. retroflexus* in the Czech Republic to terbuthylazine and metamitron was robustly confirmed using dose–response tests, chlorophyll fluorescence measurements, and molecular analysis. Notably, biotypes R1 and R2 exhibited significantly high GR50 and RF values in terbuthylazine tests, signaling target-site resistance. Genetic studies aligned with these findings, showing the widespread S264G mutation in the *psbA* gene encoding the D1 protein, especially prevalent in biotype R2. In biotype R1, fewer plants displayed resistance characteristics, and only 40% harbored the S264G mutation. Chlorophyll fluorescence, particularly the QY PSII measurement, proved consistent and efficient, suggesting its utility for field assessments. Despite the mutation’s impact on terbuthylazine resistance, no change in binding patterns with metamitron was detected, underscoring the mutation’s specificity. In summary, the S264G mutation underpins terbuthylazine and metamitron resistance in Czech *A. retroflexus*, with future studies needed to unravel the detailed resistance mechanisms. The influence of climate change on herbicide efficacy underscores the need for adaptive integrated weed management strategies.

## Figures and Tables

**Table 1:** The increasing doses of three active ingredients used in the dose–response tests.

Active Ingredient (a.i)	0.01 N	0.0316 N	0.1 N	0.316 N	1 N	3.16 N	10 N	31.6 N
terbuthylazine	7.5	23.7	75	237	750	2370	7500	23,700
metamitron	14	44.24	140	442.4	1440	4424	14,000	44,240

N—recommended field dose in the Czech Republic, x N—dose multiples.

**Table 2:** Calculated GR50 (g a.i. ha^−1^) and RF values from dose–response assay for R1, R2, and the reference sensitive biotypes (S). The letters indicate homogeneous groups at 5% significance level.

Herbicide	Biotype	GR50 (g a.i. ha^−1^) ^1^	CI ^2^	RF ^3^
terbuthylazine	R1	5512.5	4081.5–6943.5	120.49
	R2	>23,700	-	>518.032
	S	45.75	33.75–56.25	-
metamitron	R1	3617.6 ^1^	3362.8–3871	3.275
	R2	4825.8	4142.6–5511.8	4.369
	S	1104.6	1104.6–1020.6	-

^1^ GR50, growth reduction, ^2^ CI, confidence interval (95%), ^3^ RF, resistance factor.

**Table 3:** 3D molecular modeling analysis of the *A. retroflexus* D1 protein.

Protein Name	Template	Sequence Identity	Description	GMQE	QMEANDisCoGlobal	Ramachandran Favored
Wild type	4yuu.1.A	89.24%	Crystal structure of oxygen-evolving photosystem II from a red alga	0.84	0.75 ± 0.05	96.49%
S264G	4yuu.1.A	88.95%	Crystal structure of oxygen-evolving photosystem II from a red alga	0.84	0.74 ± 0.05	96.49%

## Data Availability

The raw data supporting the conclusions of this article will be made available by the authors on request.

## Supplementary Materials

The following supporting information can be downloaded at: https://www.mdpi.com/article/10.3390/genes15070904/s1, Figure S1: Changes in Fv/Fm and effective quantum yield of PS II in 20 plants of R1, R2, and S biotype after the application of terbuthylazine; Figure S2: Changes in Fv/Fm and effective quantum yield of PS II in 20 plants of R1, R2, and S biotype after the application of metamitron.
