# Supplementary material for: Molecular Mechanisms of Resistance against PSII-Inhibiting Herbicides in Amaranthus retroflexus from the Czech Republic
Source: Genes (Basel). 2024 Jul 11;15(7):904. doi: 10.3390/genes15070904 (PMC11275581; doi:10.3390/genes15070904)
Supplement: Supplementary file 1 [file genes-15-00904-s001.zip › genes-3049922-supplementary.pdf]

Appendix A

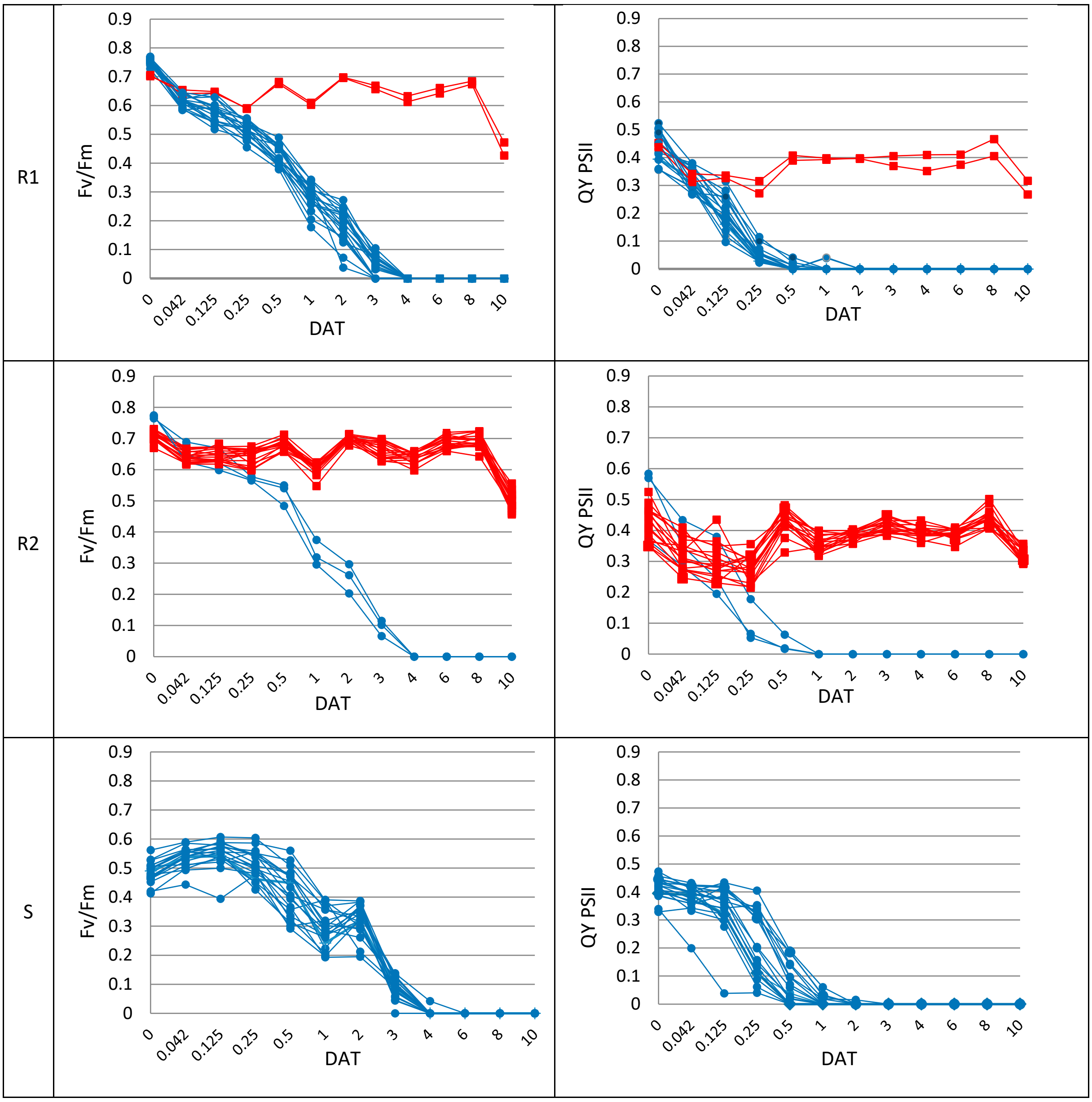

Figure A1. Changes in Fv/Fm and effective quantum yield of PS II in 20 plants of R1, R2 and S biotype after the application of terbuthylazine.

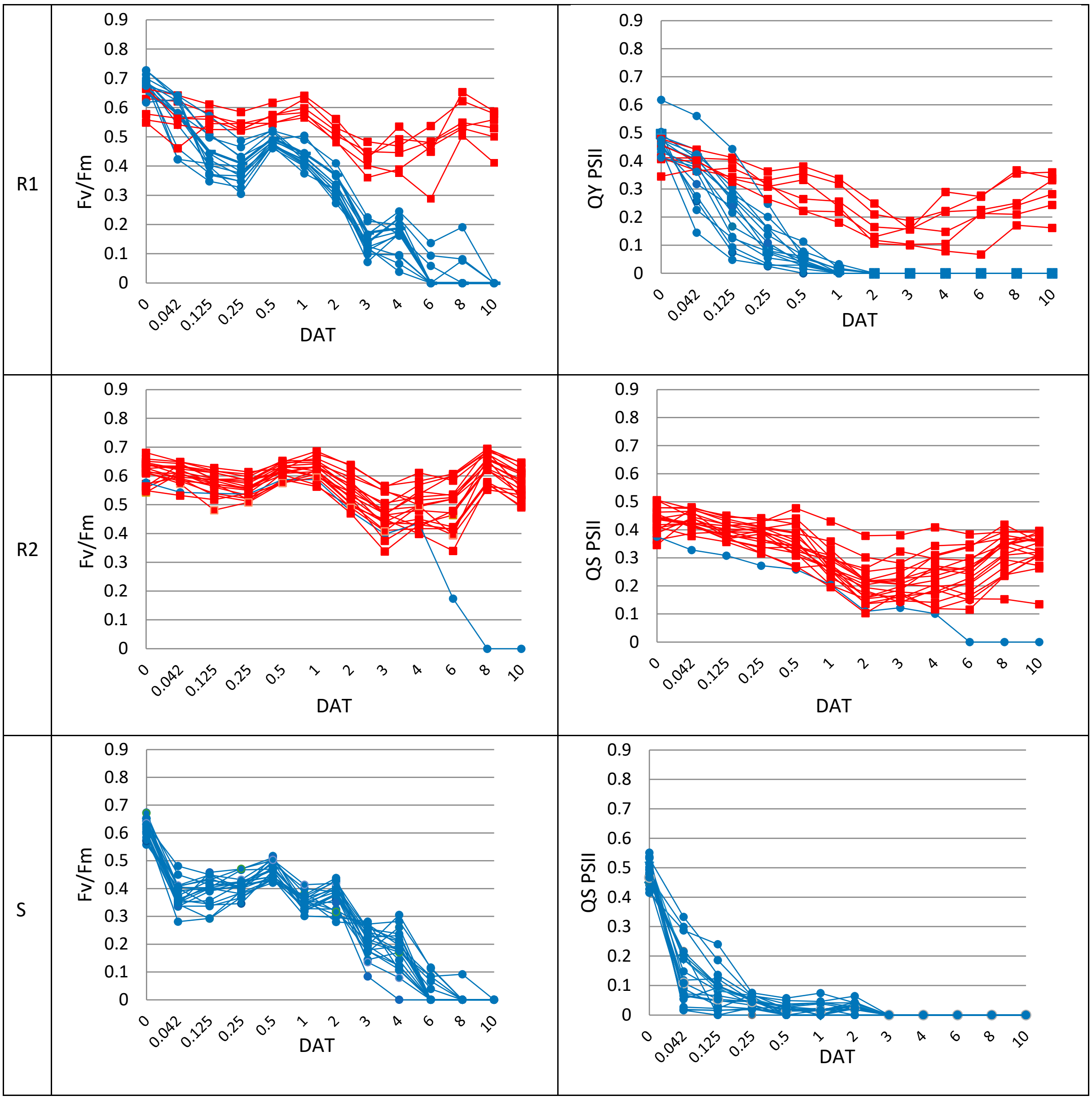

Figure A2. Changes in Fv/Fm and effective quantum yield of PS II in 20 plants of R1, R2 and S biotype after the application of metamitron.
